# Geographic distribution of echinococcosis in Tibetan region of Sichuan Province, China

**DOI:** 10.1186/s40249-018-0486-4

**Published:** 2018-11-02

**Authors:** Lei Liu, Bing Guo, Wei Li, Bo Zhong, Wen Yang, Shu-Cheng Li, Qian Wang, Xing Zhao, Ke-Jun Xu, Sheng-Chao Qin, Yan Huang, Wen-Jie Yu, Wei He, Sha Liao, Qi Wang

**Affiliations:** 1 Department of Parasitic Diseases, Sichuan Center for Disease Control and Prevention, No.6 Zhongxue Road, Chengdu, 610041 China; 2 West China School of Public Health, Sichuan University, No.16 3rd Section of Renmin South Road, Chengdu, 610041 China; 3 Ganzi Center for Disease Control and Prevention, No.139 Lucheng South Road, Ganzi Prefecture, 626000 China; 4 Aba Center for Disease Control and Prevention, No.178 Meigu Street, Aba Prefecture, 624000 China

**Keywords:** Echinococcosis, Spatial autocorrelation analysis, Moran’s *I*, Spatial cluster analysis

## Abstract

**Background:**

Echinococcosis is a parasitic zoonosis caused by *Echinococcus* larvae parasitism causing high mortality. The Tibetan Region of Sichuan Province is a high prevalence area for echinococcosis in China. Understanding the geographic distribution pattern is necessary for precise control and prevention. In this study, a spatial analysis was conducted to explore the town-level epidemiology of echinococcosis in the Sichuan Tibetan Region and to provide guidance for formulating regional prevention and control strategies.

**Methods:**

The study was based on reported echinococcosis cases by the end of 2017, and each case was geo-coded at the town level. Spatial empirical Bayes smoothing and global spatial autocorrelation were used to detect the spatial distribution pattern. Spatial scan statistics were applied to examine local clusters.

**Results:**

The spatial distribution of echinococcosis in the Sichuan Tibetan Region was mapped at the town level in terms of the crude prevalence rate, excess hazard and spatial smoothed prevalence rate. The spatial distribution of echinococcosis was non-random and clustered with the significant global spatial autocorrelation (*I* = 0.7301, *P* = 0.001). Additionally, five significant spatial clusters were detected through the spatial scan statistic.

**Conclusions:**

There was evidence for the existence of significant echinococcosis clusters in the Tibetan Region of Sichuan Province, China. The results of this study may assist local health departments with developing better prevention strategies and prompt more efficient public health interventions.

**Electronic supplementary material:**

The online version of this article (10.1186/s40249-018-0486-4) contains supplementary material, which is available to authorized users.

## Multilingual abstracts

Please see Additional file 1 for translations of the abstract into the five official working languages of the United Nations.

## Background

Echinococcosis is one of the parasitic zoonoses caused by *Echinococcus* larvae parasitizing humans or animals. Humans are infected by eating worm eggs discharged from the intestinal tract of the definitive host (canid). In the human body, all organs can be affected by echinococcosis, especially the liver, lung and brain, which present occupying lesions; additionally, systemic or local allergic reactions can occur if the cysts rupture [1–3]. In China, two types of hydatid disease (cystic echinococcosis [CE] and alveolar echinococcosis [AE]) exist within the endemic region, which includes the northwest pastoral area and Tibetan Plateau [4, 5]. According to the WHO, Alveolar echinococcosis is called “parasitic cancer” because it causes a high mortality (> 90%) among patients who do not receive sustained treatment [6, 7].

The Tibetan Region of Sichuan Province, including Ganzi Prefecture, Aba Prefecture, parts of Liangshan Prefecture and Ya’an City, has one of the highest prevalence rates of echinococcosis in China. According to previous surveys, almost all cases in Sichuan Province are concentrated in this area [8, 9]. In 2012, a large-scale epidemiological survey was conducted and obtained an overall prevalence rate as high as 1.15%. A total of 12 counties were defined as high endemic areas with prevalence rates greater than 1%, and 14 counties were proven to be mixed endemic areas with a prevalence of both CE and AE [10].

Spatial epidemiology, which is an important branch of epidemiology and geography, utilizes geographic information systems and spatial analysis techniques to describe spatial distribution characteristics and analyse changes and differences in the geographical distributions of diseases. This field explores the specific influencing factors and provides strategies and references for disease control and prevention. In recent years, many spatial epidemiological studies have focused on the regional distribution of echinococcosis with an aim of detecting high prevalence areas. To the best of our knowledge, most of these studies have been conducted in single administrative divisions and have neglected spatial distribution pattern analysis. In theory, relativity may exist among cases in adjacent areas due to homogeneity of the infection source and transmission path, such as the number of host animals and geographical features.

Obtaining a better understanding of the spatial distribution patterns of echinococcosis could help identify populations at high risk, explore influencing factors and provide guidance for more accurate control strategies. Geographic information system (GIS) software combined with spatial analysis methods provides powerful tools to characterize spatial patterns of diseases. Spatial autocorrelation analysis can be performed to detect a significant difference from a random spatial distribution [11–13]. In addition, spatial cluster analysis can be conducted to identify whether cases of the disease are geographically clustered [14–16].

In this study, we conducted a GIS-based spatial analysis involving spatial smoothing and spatial clustering analysis to characterize the geographic distribution patterns of echinococcosis cases in the Tibetan Region of Sichuan Province.

## Materials and methods

### Study area and data sources

The Sichuan Tibetan Region is located in the southeast Qinghai-Tibet Plateau (east longitude 97°26′–104°27′, north latitude 27°57′–34°21′), which borders Qinghai and Gansu provinces in the north, Tibet Autonomous Region in the west, Yunnan Province in the south and Sichuan Basin in the southeast. This region covers an area of 249 700 km^2^ and has a population of 2 million.

Data from echinococcosis cases were obtained from screening reports up to the end of 2017. A general survey was conducted with community residents according to the diagnostic criteria of hydatid disease by local medical institutions over the past two years [17]. Information was collected from all patients and summarized at the town level based on address information.

Population data from every town were retrieved from the National Bureau of Statistics of China. To conduct a GIS-based spatial distribution analysis, we obtained the town-level polygon map at the 1:100000 scale, on which the town-level point layer containing information regarding latitudes and longitudes of central points for each town was created. All cases were geo-coded and matched to the town-level layers of the polygon and point by administrative codes using the free open-source software R (version 3.4.3. Bell Laboratories, New Jersey, United States), which is a free software programming language and a software environment for statistical computing and graphics. We used the SP package for spatial visualization [18, 19].

### GIS mapping and Bayes smoothing

Echinococcosis cases per 100 individuals in each town were calculated to alleviate variations in the prevalence rates in small populations and areas. To assess the risk of echinococcosis in each town, an excess hazard map was produced. The excess hazard represented the ratio of the observed prevalence in each town over the average prevalence of all towns; the average prevalence was calculated as the number of cases over the total number of people at risk. The excess hazard was equal to the standardized morbidity ratio (SMR), which is the ratio of the observed cases to the expected cases of echinococcosis within a town. Then, spatial empirical Bayes smoothing was implemented by the DCluster package in R to make the SMR more stable [20].

### Spatial autocorrelation analysis

Moran’s *I* statistic is one of the most commonly used measures for spatial autocorrelation. In this study, Assuncao and Reis’s global Moran’s *I* statistic was applied to discern spatial autocorrelation and detect the spatial distribution pattern of echinococcosis in the Sichuan Tibetan Region after adjusting for population heterogeneity [21, 22]. The statistic was calculated as follows, and the analyses were performed in the spdep package in R.

$$ I=\frac{n{\sum}_i{\sum}_j{W}_{ij}\left({X}_i-\overline{X}\right)\left({X}_j-\overline{X}\right)}{\sum_i{\sum}_j{W}_{ij}{\left({X}_i-\overline{X}\right)}^2} $$

where:

*X*_*i*_ = the crude prevalence rate for the *i*th town;

$\overline{X}$ = the mean crude prevalence rate for all towns;

*X*_*j*_ = the crude prevalence rate for the *j*th town;

*W*_*ij*_ = a weight parameter for the pair of towns *i* and *j* that represents proximity; and.

*n* = the number of towns.

*I* > 0 indicates a clustered pattern, *I* = 0 indicates a random pattern, and *I* < 0 indicates a dispersed pattern.

### Spatial cluster analysis

Spatial scan statistical analysis was performed to examine the local clusters using the SaTScan software (v9.4.4. Management Information Services, Maryland, United States). In this method, a circular window was imposed with a varied centroid and a flexible radius from zero to some upper limit, and a large number of distinct geographical circles were created with different sets of neighbouring data locations within them. Each circle was a possible candidate cluster. The null hypothesis assumed that the relative risk (RR) was the same both within and outside of the window. A Poisson-based model was used in which the number of events in an area exhibited a Poisson distribution according to a known underlying population at risk. The significance test of the identified clusters was calculated by comparing the log likelihood ratio (LLR) test statistics against a null distribution obtained from the Monte Carlo simulation [15, 23]. The number of permutations was set to 999, and the level of significance was set at *P* < 0.05.

$$ LLR(z)=\ln \frac{L(z)}{L0}={\left(\frac{cz}{nz}\right)}^{cz} $$

where:

*c*_*z*_ = the observed number of patients in each circle unit;

*C* = the total number of patients in the study region; and.

*n*_*z*_ = the expected number of patients in each circle unit.

## Results

### Descriptive analysis of echinococcosis cases

**Fig. 1:**
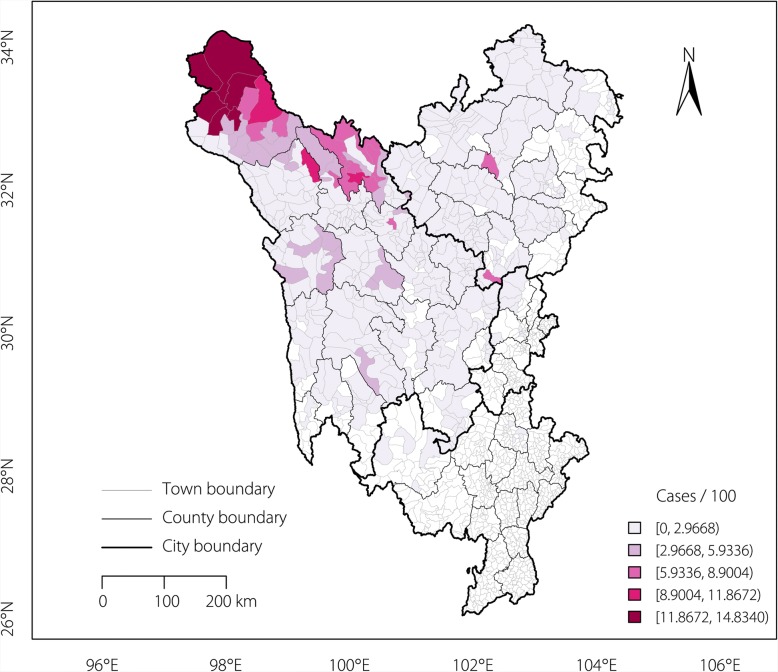
Prevalence rates of echinococcosis at the town level in the Sichuan Tibetan Region. The prevalence rates were calculated by cases of echinococcosis per 100

A total of 14 063 echinococcosis cases were detected in the Sichuan Tibetan Region by the end of 2017. The prevalence rates at the town level ranged from 0 to 14.83 per 100 (Fig. 1). The towns with the highest prevalence were mainly concentrated in the northwest region.

**Fig. 2:**
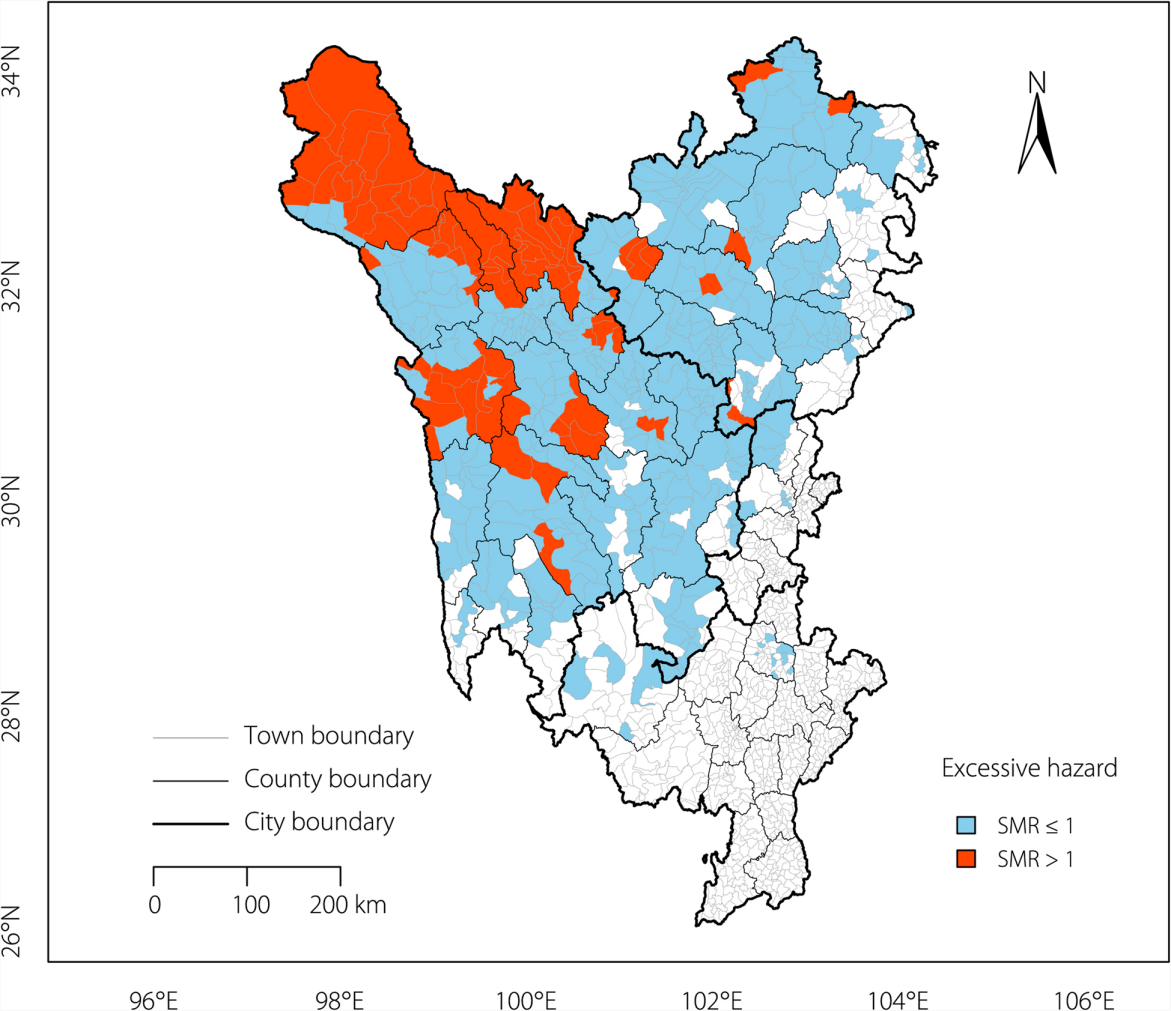
Excess hazard map of echinococcosis in the Sichuan Tibetan Region. Towns shown in a sky-blue colour had lower prevalence rates than expected (SMR less than 1), whereas towns shown in an orange-red colour had higher prevalence rates than expected (SMR greater than 1)

**Fig. 3:**
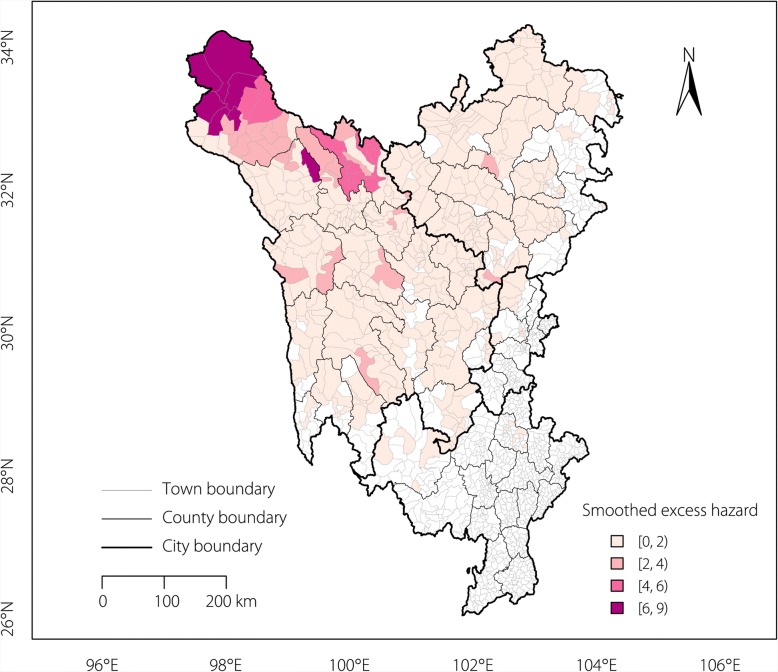
Spatially smoothed excess hazard map of echinococcosis in the Sichuan Tibetan Region. SMR: Standardized morbidity ratio

The excess hazard map (Fig. 2) showed the distribution of the excess risk, which was defined as a ratio of the observed number over the expected number of cases. Towns shown with a sky-blue colour had lower prevalence rates than expected, as indicated by a SMR less than 1. In contrast, towns shown with an orange-red colour had higher prevalence rates than expected or a SMR greater than 1. A spatial empirical Bayes smoothed map for excess hazards (Fig. 3) was created by correcting the variance in the population variability.

### Global spatial autocorrelation and cluster analysis

The global spatial autocorrelation analyses for echinococcosis cases in the Sichuan Tibetan Region showed that Moran’s *I* was statistically significant (*I =* 0.7301, *P* = 0.001), implying that the distribution of echinococcosis was spatially autocorrelated.

**Fig. 4:**
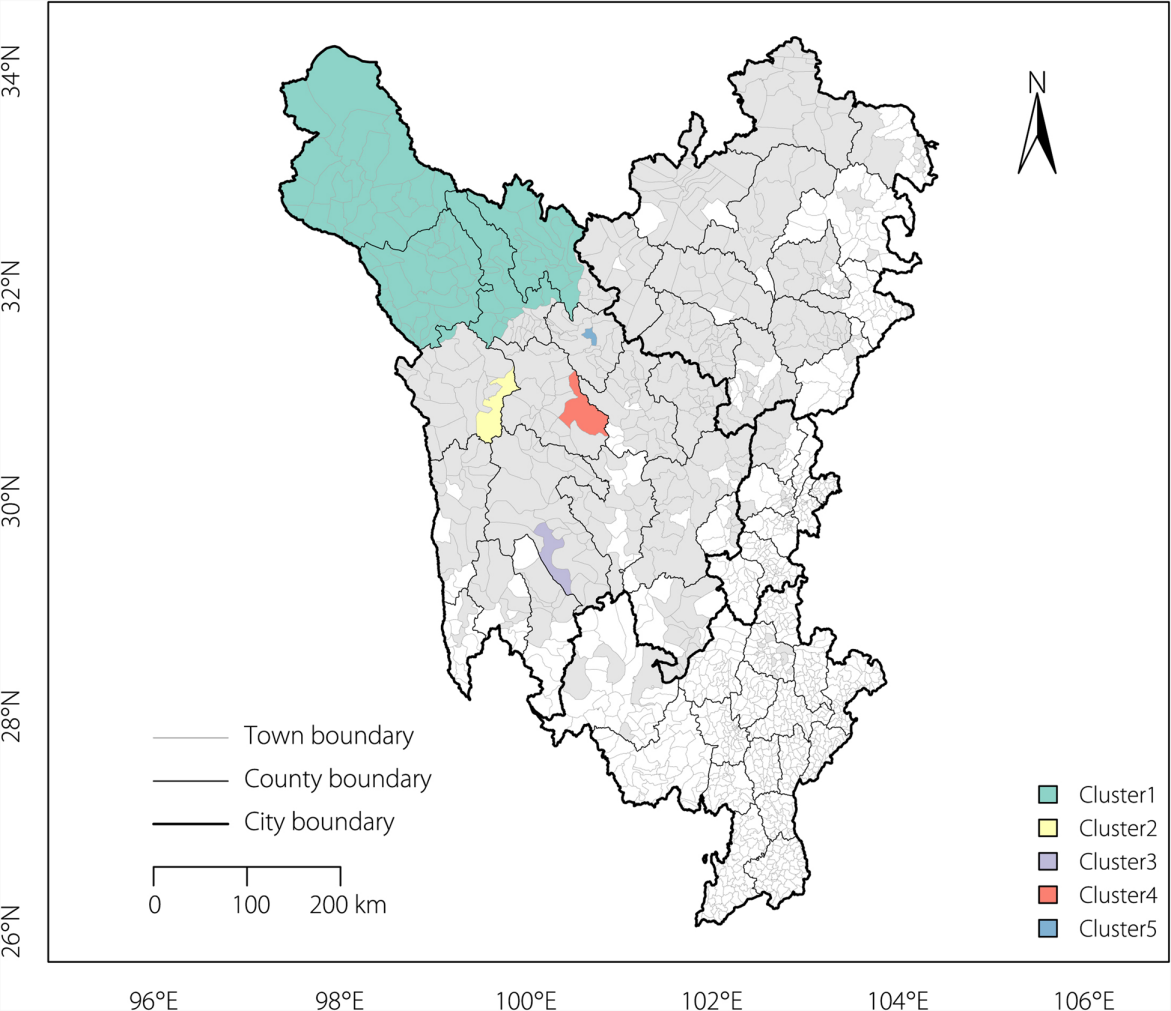
Spatial clusters (hotspots) of echinococcosis in the Sichuan Tibetan Region

Using a maximum spatial size of 50% of the total population, five significant spatial clusters (hot spots) of echinococcosis cases were identified via the spatial scan statistics (Table 1 and Fig. 4). The most likely cluster for high prevalence was in the northwest and covered Shiqu County, Seda County, Dege County and Ganzi County.

**Table 1:** Spatial clusters of echinococcosis in the Sichuan Tibetan Region

Cluster	Centre coordinates	Number of cases	Expected cases	Relative risk	*P* value
1	32.900478 N, 99.098418 E	10 614	3392.56	9.75	< 0.0001
2	30.858954 N, 99.673140 E	132	44.21	3.00	< 0.0001
3	29.483547 N, 100.295973 E	56	20.89	2.69	< 0.0001
4	30.835933 N, 100.620987 E	81	36.65	2.22	< 0.0001
5	31.538112 N, 100.699541 E	23	5.51	4.18	0.00012

## Discussion

In our study, an exploratory spatial autocorrelation analysis and spatial cluster analysis of echinococcosis were conducted at the town level in the Sichuan Tibetan Region, China. We mapped echinococcosis based on different aspects, including the crude prevalence, excess risk, and spatial empirical Bayes smoothed prevalence, and investigated the spatial patterns and highlighted geographic areas with significantly high prevalence rates. Spatial epidemiology is a discipline that combines geography, epidemiology, statistics and demography with a focus on spatial distribution patterns and spatial clusters. This field helps researchers understand the geographic distribution and incidence trend of diseases, thereby providing theoretical guidance for disease surveillance and preventive measures. Studies of spatial distribution patterns aim to clarify the diversity and variability of a disease distribution in a geographical space. Spatial clustering refers to areas in which the risk of disease is significantly higher than that in other areas as a result of the presence of infectious disease pathogenic factors or potential risk factors. Exploring the characteristics of spatial epidemiology has great importance for the prevention and control of echinococcosis. The spatial statistics provide a methodological basis for the identification of spatial clusters by showing “hot spots” and “cold spots”. Moreover, Moran’s *I* index is one of the most commonly used spatial autocorrelation analysis methods and demonstrates the overall spatial pattern. The two methods are often used together.

Although similar studies have been conducted, generally they have been limited to separated administrative districts. Therefore, observing correlations among neighbouring districts is difficult [24–26]. Compared with these previous studies, the current study covered all Tibetan counties in Sichuan Province simultaneously and revealed a large scale of spatial characteristics of the disease that facilitated an integrated analysis of the potential risk factors of spatial aggregation in multiple districts.

In addition, our research was based on the township level, which was not assessed in previous studies. The finer the division of the study area, the more accurate the conclusion of the spatial analysis. However, when the study population is small, the direct calculation of the prevalence is not stable, and revealing real high-prevalence regions is difficult due to substantial noise. Hence, spatial empirical Bayes smoothing was applied to account for the heterogeneity of population sizes between townships. The principle of Bayesian modelling inferred the unknown parameters by synthesizing the overall and regional information to derive the posterior distribution of each township according to the Bayesian formula. The empirical Bayesian smoothing was based on Bayesian statistics and was applied to the smooth calculation of various small-probability events with the purpose of obtaining a more stable prevalence rate through statistical adjustment according to the principle that the large population rate was more stable than that of the small population [27, 28].

From an overall perspective, the epidemical situation of echinococcosis in Sichuan Tibetan Region was serious, especially in some northwest counties, and the prevalence rates were startling. The high prevalence was related to local production and living habits. *Echinococcus* requires two mammalian hosts to complete its life cycle. The definitive host (carnivore) passes eggs in the faeces. The eggs are ingested by the intermediate host, in which the metacestode stage and protoscoleces develop. The cycle can be completed if the *Echinococcus* in the intermediate host is eaten by a carnivore [1]. In the Sichuan Tibetan Region, grassland accounts for the majority of the area, and thus most of the local Tibetan residents herd livestock (yak and sheep) for a living. At the same time, large numbers of dogs are fed to protect against wild animal attacks. Human factors allow interaction between the natural and domestic cycles and have resulted in widespread perpetuation of *Echinococcus* in man-made life cycle pattern. In addition, local residents have poor hygiene practices due to the low level of economic development, such as feeding dogs slaughtered cattle offal, not washing their hands after playing with the dogs, and using their hands to make and eat tsamba, which has also led to the growing number of patients [29–32].

The results showed that the spatial distribution of echinococcosis in the Sichuan Tibetan Region was non-random and clustered with a significant Moran’s *I*. Five significant spatial clusters with high prevalence rates were detected using spatial scan statistics. According to the spatial distribution maps, most cases and clusters were concentrated in the northwest counties. This finding was consistent with the distribution of the primary infection source (i.e., carnivores). In the northwest counties, the size of the grassland accounted for a larger proportion of terrain classification than in other Tibetan counties, and the ratio of owned and stray dogs was also higher. Large number of dogs and other wild host animals were widely distributed in these districts, which introduced a greater risk of human infection. Moreover, the infections in animal hosts were also relatively high in these counties based on surveillance data. In the four counties comprising the largest cluster (Shiqu, Seda, Dege and Ganzi), the *Echinococcus* infection rates in dogs ranged from 2.29 to 3.92%, which were significantly higher than the provincial average of 1.68%. In addition, the northwest counties belong to the typical Qinghai-Tibet Plateau landform with high altitudes and a cold climate. Harsh natural conditions, poor sanitary facilities, little health awareness, bad living habits, and insufficient preventive measures further increased infections in local residents.

Over last ten years, a variety of nationally and provincially supported intervention strategies and measures have been implemented in the Sichuan Tibetan Region, including medication and surgical treatment for patients, monthly praziquantel treatment for dogs, slaughter management for livestock, and public health education. These measures have effectively blocked the chain of transmission and curbed the emergence of new cases [33]. In November 2015, the comprehensive prevention pilot project on echinococcosis launched in Shiqu County. A combination of preventive measures was conducted, including patient screening for the whole population, surgical treatment and medical therapy, dog standardized management, slaughter management and deworming of domestic animals, various health education initiatives, and ensuring a safe drinking water supply. After two years of comprehensive prevention and management, the transmission of *Echinococcus* was effectively controlled, the rate of new patients decreased yearly, and the new mode of control was developed and promoted.

The results of the study provided useful information on the prevailing epidemiological situation for echinococcosis in the Sichuan Tibetan Region. The novel discovery of clusters can help administrative agencies and disease prevention institutions intensify their remedial measures in the identified areas with a high prevalence and delineate future strategies for more effective control. The results provided scientific references and recommendations for further decision-making and allocation of medical resources. Investing more efforts in the high-prevalence and clustered areas will be more cost-effective. In addition, our research methods, especially spatial autocorrelation and spatial scanning, can provide references for other similar studies.

The prevalence of echinococcosis in the Sichuan Tibetan Region indicates a major disease that causes poverty due to the loss of labour capacity or high surgical costs. Defining the spatial distribution of this disease is urgent for prevention and control. Further combination with the application of geographic information systems, global positioning systems and spatial statistical methods should be applied to comprehensively investigate the spatial epidemiology of echinococcosis.

Our study has several limitations. Currently, the echinococcosis monitoring system in the Sichuan Tibetan Region is not complete and integral, and some counties lack animal host infection status data at the town level. In the future, we need to expand the monitoring scope and explore the relationship between human prevalence and animal infection.

In the cluster analysis, Qinghai, which is located in the northwest Sichuan Tibetan Region bordering Tibet, showed an extremely high prevalence rate. This finding hinted that the cluster area might cover a greater scope and prompted us to conduct a broader epidemiological analysis with joint multiple administrative regions.

## Conclusions

This study explored the spatial distribution patterns of echinococcosis in the Sichuan Tibetan Region. The spatial distribution is non-random and clustered with significant global spatial autocorrelation in the area, and spatial clusters (hot spots) of echinococcosis cases are identified via spatial scan statistics. According to the spatial distribution maps, most of the cases and clusters are concentrated in the northwest counties of the Sichuan Tibetan Region, which is directly related to the distribution of infectious sources, human behaviour and natural environmental factors. In recent years, a series of intervention strategies and measures have been implemented and have achieved good results. On this basis, the spatial analysis could supply more useful information about the prevalence situation of echinococcosis and thus enhance and improve the existing strategies to control the disease. The results of this study will assist local health departments with developing better preventive methods and prompt more efficient public health interventions. The results may also stimulate further ecological and pathogenic biological research on the possible causes underlying the clusters and spatial associations of echinococcosis in the Tibetan area of Sichuan and its adjacent areas.

## Additional file

Additional file 1: Multilingual abstracts in the five official working languages of the United Nations. (PDF 227 kb)

## Abbreviations

AE: Alveolar echinococcosis
CE: Cystic echinococcosis
LLR: Log likelihood ratio
RR: Relative risk
SMR: Standardized morbidity ratio
WHO: World Health Organization
