# Supplementary material for: Geographic distribution of echinococcosis in Tibetan region of Sichuan Province, China
Source: Infect Dis Poverty. 2018 Nov 2;7:104. doi: 10.1186/s40249-018-0486-4 (PMC6214160; doi:10.1186/s40249-018-0486-4)
Supplement: Supplementary file 1 — Multilingual abstracts in the five official working languages of the United Nations. (PDF 227 kb) [file 40249_2018_486_MOESM1_ESM.pdf]

Translation of the abstract into the five official working languages of the United Nations

التوزيع الجغرافي لمرض المشوكات في منطقة التبت بمقاطعة سيتشوان، الصين

لى ليو [†1], بينج جوا [†2], وي لى [†3], بو جونج [*1], وين يانج [1], شو- تشنج لي، تشيان وانج ، شينج تشاو ، كه-جون شو، شينج-تشاو تشين، يان هوانج ، وين-جي يو ، وي هي ، شا لياو ، تشى وانج

الملخص
الخلفية:داء المشوكات من الأمراض الحيوانية الطفيلية ذو معدل وفيات مرتفع وينتقل عن طريق يرقات ديدان طفيلية. تعتبر منطقة التبت في مقاطعة سيتشوان ذات انتشار واسع لمرض المشوكات في الصين . لذلك ، من الضروري فهم نمط التوزيع الجغرافي من أجل مكافحة المرض بدقة والوقاية منه. في هذه الدراسة ، تم إجراء تحليل مكاني لاستكشاف التوزيع المكاني لوباء مرض المشوكات في منطقة ستيشوان التبتية وتوفير التوجيهات اللازمة لصياغة استراتيجيات الوقاية والمكافحة الإقليمية.
المنهج: استندت الدراسة إلى حالات الإصابة بالمشوكات التي تم الإبلاغ عنها في نهاية عام 2017 ، وتم ترميز كل حالة جغرافياً على مستوى المدينة. تم الاستعانة بمقدر بايز الاعتباري للتمهيد المكاني والارتباط الذاتي المكاني العالمي للكشف عن نمط التوزيع المكاني . تم تطبيق احصائيات المسح المكاني لفحص التجمعات المحلية.
النتائج: تم تخطيط التوزيع المكاني لمرض المشوكات في منطقة سيتشوان التبتية على مستوى البلدة من حيث معدل الإنتشار التقريبي والخطر الزائد ومعدل الانتشار المكاني الممهد. كان التوزيع المكاني لمرض المشوكات غير عشوائي وتم تجميعه مع الترابط الذاتي المكاني العالمي (I = 0.7301, P = 0.001). بالإضافة إلى ذلك ، تم الكشف عن خمس مجموعات مكانية كبيرة من خلال احصاء المسح المكاني.
الاستنتاج: هناك دليل على وجود مجموعات كبيرة من طفيل المشوكة في منطقة التبت بمقاطعة سيتشوان الصينية. قد تساعد نتائج هذه الدراسة دوائر الصحة المحلية على تطوير استراتيجيات وقائية أفضل وإحداث تدخلات أكثر فعالية في مجال الصحة العامة.

Translated from English version into Arabic by Free bird and Heba Kandel, through

TRANSLATORS
WITHOUT BORDERS

中国四川藏区包虫病地理分布研究

刘磊，郭冰，李伟，钟波，杨文，李树成，王谦，赵星，徐克均，秦胜超，黄燕，喻文杰，何伟，廖沙，王奇

摘要
引言：包虫病是由棘球绦虫的幼虫寄生于人体引起的一种人畜共患病，死亡率很高。四川省藏区是中国包虫病的高发地区，了解该区域内包虫病的地理分布模式对于精确控制和预防是必要的。本研究采用空间分析方法，探索了四川藏区包虫病在乡镇水平上的流行病学特征，为制定该区域的防控策略提供科学依据。
方法：本研究基于截止到 2017 年底的包虫病病例，在乡镇水平对病例进行地理编码。运用空间经验贝叶斯平滑和全局空间自相关探索疾病的空间分布模式，应用空间扫描统计分析来探测病例的聚集性。
结果：在城镇水平上，通过粗患病率、超额患病率和空间平滑患病率展示了四川藏区的包虫病空间分布。研究区域内包虫病的空间分布是非随机的，并且具有显着的全局空间自相关性

（I = 0.7301，P = 0.001）。此外，通过空间扫描统计探测了五个空间聚集区。
结论：包虫病在中国四川藏区的分布存在明显的空间聚集性。此研究的结果将有助于当地卫生部门制定更好的预防策略，并促进更有效的公共卫生干预措施。

Translated from English version into Chinese by Lei Liu

Distribution géographique de l'échinococcose dans la région tibétaine de la province du Sichuan, en Chine

Lei Liu, Bing Guo, Wei Li, Bo Zhong, Wen Yang, Shu-Cheng Li, Qian Wang, Xing Zhao, Ke-Jun Xu, Sheng-Chao Qin, Yan Huang, Wen-Jie Yu, Wei He, Sha Liao, Qi Wan

Résumé
Contexte : l'échinococcose est une zoonose parasitaire causée par les larves du parasite *Echinococcus*, présentant un taux de mortalité élevé. La région tibétaine de la province du Sichuan est une région de la Chine où l'échinoccose est très fréquente. Il est ainsi nécessaire de comprendre les modèles de distribution géographique afin d'assurer une prévention et un contrôle précis. Dans l'étude suivante, une analyse spatiale a été menée dans le but d'explorer l'épidémiologie spatiale de l'échinoccose dans la région tibétaine de la province du Sichuan et de fournir des conseils en matière de prévention régionale et de stratégies de contrôle.
Méthodes : l'étude est basée sur des cas d'échinoccose signalésavant la fin 2017, et chaque cas a été géocodé à l'échelle municipale. Le lissage spatial empirique de Bayes et l'autocorrélation spatiale globale ont été utilisées afin d'identifier un modèle de distribution spatiale. Une analyse spatiale des statistiques a été appliquée afin d'examiner les grappes locales.
Résultats :la distribution spatiale de l'échinoccose dans la région tibétaine du Sichuan a été cartographiée à une échelle municipale en termes de taux brut de prévalence, de risques excessifs et de taux spatial lissé de prévalence. La distribution spatiale de l'échinoccose s'est révélée non aléatoire et regroupée selon l'autocorrélation spatiale globale significative (I = 0,7301, P = 0,001). De plus, cinq grappes spaciales significatives ont été détectées grâce à l'analyse spatiale des statistiques.
Conclusions : de nombreuses preuves ont confirmé l'existence de grappes significatives de l'échinoccose dans la région tibétaine du Sichuan, en Chine. Les résultats de cette étude pourraient s'avérer utiles pour les services sanitaires locaux afin de développer de meilleures stratégies de prévention et de susciter des interventions de santé publique plus efficaces.

Translated from English version into French by Louis Gauvreau and Cathy Davies, through

География распространения эхинококкоза в Тибетском районе провинции Сычуань (Китай)

Лэй Лю, Бин Го, Вэй Ли, Бо Чжун, Вэнь Ян, ШуШу--Чэн Ли, Цянь Ван, Син Чжао, Кэ-Цзюнь Сюй, Шэн-Чао Цинь, Янь Хуан, Вэнь-Цзе Юй, Вэй Хэ, Ша Ляо, Ци Ван

Аннотация

Общие сведения: Эхинококкоз – это паразитарный зооноз , вызываемый личинками *Echinococcus* , с высоким уровнем смертности. В Китае эхинококкоз широко распространен в Тибетском районе провинции Сычуань. Поэтому для эффективного контроля и предотвращения эхинококкоза необходимо уточнить географию его распространения. В ходе данного исследования был проведен географический анализ распространения эхинококкоза в Тибетском районе провинции Сычуань, на основе которого могут быть разработаны региональные стратегии предотвращения и контроля за распространением этого заболевания.
Методы: Исследование было основано на случаях эхинококкоза, зарегистрированных на конец 2017 года; каждому случаю был присвоен геокод на уровне городов. С помощью пространственного эмпирического байесовского сглаживания и глобальной пространственной автокорреляции была уточнена география распространенности заболевания. Для изучения локальных кластеров были применены методы пространственного сканирования.
Результаты:Простанственная распространенность эхинококкоза в Тибетском районе провинции Сычуань на уровне городов была нанесена на карту в виде общего показателя распространенности, показателя смертности от эхинококкоза и пространственного сглаженного показателя распространенности. Пространственная распространенность эхинококкоза была упорядоченной, с выделением кластеров, и имела значительную глобальную пространственную автокорреляцию (I = 0.7301, P = 0.001). Кроме того, посредством статистики пространственного сканирования было выявлено пять больших пространственных кластеров.
Выводы: Были обнаружены доказательства наличия значительных кластеров эхинококкоза в Тибетском районе провинции Сычуань (Китай). Результаты данного исследования могут помочь местным отделам здравоохранения в усовершенствовании стратегии профилактики и способствовать более эффективному вмешательству в области здравоохранения.

Translated from English version into Russian by Daria and Alexander Somin, through

TRANSLATORS
WITHOUT BORDERS

Distribución Geográfica de Equinococosis en la Región Tibetana de la Provincia de Sichuán, China.

Lei Liu[1†], Bing Guo[2†], Wei Li[3†], Bo Zhong[1*], Wen Yang[1], Shu-Cheng Li, Qian Wang, Xing Zhao, Ke-Jun Xu, Sheng-Chao Qin, Yan Huang, Wen-Jie Yu, Wei He, Sha Liao, Qi Wang

Resumen

Contexto: Equinococosis es una zoonosis parasitaria causada por *la larva Equinococo* de alta mortalidad. La región tibetana de la provincia de Sichuán es una zona de alta propagación de la equinococosis en China. Por lo tanto, es necesario entender el patrón de distribución geográfico para precisar el control y la prevención. En este estudio se realizó un análisis de la zona para explorar la epidemiología espacial de la equinococosis en la región tibetana de Sichuán y para proporcionar pautas para la prevención regional y las estrategias de control.

Métodos: el estudio se basó en los casos de equinococosis notificados a finales de 2017, y cada caso fue geocodificados a nivel local. Se utilizó la transferencia de Bayes empíricos espaciales y la autocorrelación espacial global para identificar el patrón de distribución espacial. Se aplicaron estadísticas de escaneo espacial para examinar grupos locales.

Resultados: se mapeó la distribución espacial de equinococosis en la región tibetana de Sichuán a nivel local en términos de tasa de prevalencia bruta, riesgo excesivo y tasa de prevalencia espacial regular. La distribución espacial de equinococosis no fue aleatoria y se juntó con la significativa autocorrelación espacial global (I = 0.7301, P = 0.001). Además, se detectaron cinco grupos espaciales importantes en la estadística de escaneo espacial.

Conclusiones: había evidencia de la existencia de un significante grupo de equinococosis en la región tibetana de Sichuán, China. Los resultados de este estudio pueden ayudar a los departamentos de salud locales desarrollando unas estrategias de prevención más efectivas y unas intervenciones de salud pública más rápidas y eficientes.

Translated from English version into Spanish by Sandra Mas and Constanza Olivares R, through

TRANSLATORS
WITHOUT BORDERS
